# Alcohol consumption and tobacco exposure among pregnant women in Ibadan, Nigeria

**DOI:** 10.1186/s12888-022-04210-9

**Published:** 2022-08-24

**Authors:** Ikeola A. Adeoye

**Affiliations:** 1 Department of Epidemiology and Medical Statistics, College of Medicine, University of Ibadan, Ibadan, Nigeria; 2 Consortium for Advanced Research Training in Africa (CARTA), Nairobi, Kenya

**Keywords:** Alcohol consumption, Tobacco exposure, Combined exposure, Pregnancy, Ibadan, Nigeria

## Abstract

**Background:**

Alcohol consumption and tobacco exposure during pregnancy are hazardous behaviours which are increasing significantly in low and middle-income countries, including sub-Saharan Africa. However, they have received little attention in Nigeria’s maternal health research and services. The prevalence, pattern and predictors of alcohol consumption and tobacco exposure among pregnant women in Ibadan, Nigeria, were investigated.

**Methods:**

This is a part of a prospective cohort study among pregnant women in Ibadan, Nigeria (Ibadan Pregnancy Cohort Study (IbPCS), which investigated the associations between maternal obesity, lifestyle characteristics and perinatal outcomes in Ibadan. Alcohol consumption and tobacco exposure of 1745 pregnant women were assessed during enrollment by self-reports using an interviewer-administered questionnaire. Bivariate and multiple logistic regression analyses examined the associations at a 5% level of statistical significance.

**Results:**

The prevalence of pre-pregnancy alcohol consumption and alcohol consumption during pregnancy were 551 (31.7%) and 222 (12.7%), respectively, i.e. (one in every eight pregnancies is exposed to alcohol). Palm wine (52%) and beer (12%) were the most common alcohol consumed among pregnant women. The predictors of alcohol consumption during were pre-pregnancy alcohol use [AOR = 10.72, 95% CI: 6.88–16.70) and religion i.e. Muslims were less likely to consume alcohol during pregnancy compared to Christians: [AOR = 0.60, 95% CI: 0.40–0.92). The prevalence of tobacco exposure in the index pregnancy was 64 (3.7%), i.e. one in every 27 pregnancies is exposed to tobacco. In contrast, cigarette smoking, second-hand smoke and smokeless tobacco were 0.4, 1.7 and 1.8%, respectively. Pre-pregnancy cigarette smoking was reported by 33(1.9%) and was the most significant predictor [AOR = 12.95; 95% CI: 4.93, 34.03) of tobacco exposure during pregnancy in our study population.

**Conclusions:**

Alcohol consumption and tobacco exposure are not uncommon and have been an ongoing but neglected threat to maternal and child health in Nigeria. Alcohol and tobacco control policy and programmes to prevent the use among pregnant and reproductive-age women in Nigeria should be implemented primarily during antenatal care.

## Background

Alcohol consumption and tobacco exposure during pregnancy are very harmful behaviours of public health concern because of the increasing prevalence, teratogenic effects on the fetus, adverse pregnancy outcomes, and long-term developmental impairment and disability in the child [1–4]. Alcohol is a psychoactive substance that causes intoxication, addiction, central nervous system depression and damage, all of which could cause sustained behavioural and cognitive dysfunction [5]. Alcohol during pregnancy has many detrimental effects, including adverse birth outcomes such as low birth weight, preterm birth, and small for age [5, 6]. They are also the leading causes of preventable congenital and birth defects in western countries [7]. The most burdensome of its complications is the Fetal Alcohol Spectrum Disorder (FASD), a cluster of lifelong medical conditions of varying severity caused by feotal exposure to alcohol during pregnancy [7–9]. Its manifestation includes the following: learning and cognitive disability, speech and language deficits, birth defects such as abnormal facial features, microcephaly and developmental disorders, among others [8]. Prenatal alcohol exposure has been demonstrated to impact every organ system [10], increasing mortality for diagnosed people with FASD and their siblings [11]. Also, a child with FASD has been identified as a huge marker for increased premature mortality for the mothers [12]. The long-term complications of alcohol consumption during pregnancy include mental illness, academic failure, and behavioural disorders, including substance abuse which places a considerable burden on the family, health care, educational and judicial systems [7].

Furthermore, the transgenerational effect of prenatal alcohol exposure has been reported with each episode of drinking during pregnancy exposing three generations, i.e. mother, fetus and fetal germline, to its hazardous effects [13]. Because all these complications result from preventable behaviour, the WHO and several regulatory bodies have stated that no amount of alcohol is safe in pregnancy, and pregnant women or women intending to get pregnant should abstain from alcohol [2, 6, 8]. Additionally, they have advised that there is no safe time for alcohol use during pregnancy and all types of alcohol are equally harmful, including all wines and beer.

Currently, the prevalence of alcohol intake by women of reproductive health and pregnant women is increasing in several countries, including sub-Saharan Africa. Globally, the prevalence of alcohol use in pregnancy is 9.8%, with the highest prevalence reported in Europe (25.2%) [1]. The pooled prevalence in Africa is also high, ranging from 10% [1] to 15.7% [14] with variations across the different countries; Ghana (48%) [15], South-south Nigeria (59.3%) [16] South-East Nigeria (22.6%) [17]. The risk factors for alcohol use in pregnancy include socio-economic factors like educational status, marital status, income, and employment status; obstetric factors such as complications during previous pregnancies, unplanned pregnancy, and parity; and behavioural factors like prior alcohol consumption, partner’s alcohol use, peer pressure on alcohol use, knowledge on harmful effects of alcohol use, and smoking [14]. Other factors include partner violence, urban living, current use and having a male partner who drinks alcohol, abortion history, mental distress, psychoactive substance use and lower maternal age [17, 18].

Tobacco use during pregnancy is also associated with adverse pregnancy and fetal outcomes such as ectopic pregnancy, gestational hypertension, pre-eclampsia, abruptio placentae, congenital abnormality, low birth weight, preterm birth, stillbirth, sudden infant death syndrome, and increased mortality [19–25]. Cigarette smoke contains over 4000 chemicals, including carcinogens, toxic heavy metals, teratogens and many chemicals untested for developmental toxicity [26]. The sources of tobacco exposure include cigarettes, cigars, cigarillos, roll-your-own tobacco, pipe tobacco, second-hand (environmental) tobacco smoke, smokeless tobacco, and others. Although cigarette smoking is the most common form of tobacco use worldwide, all tobacco exposure is harmful, with no safe limits to tobacco exposure during pregnancy.

Lange et al. (2018), in a systematic and meta-analysis, estimated a global prevalence of 1·7%, with much higher prevalences in the western nations (Europe 8.1%; America 5.9%) compared to other parts of the world; South-East Asia (1·2%), West Pacific (1.2%) Eastern Mediterranean (0.9%), and Africa (0.8%) [24]. Currently, sub –Saharan Africa has the lowest prevalence of tobacco during pregnancy. In Nigeria, smoking prevalence during pregnancy was 0.4% [25]. Studies investigating tobacco in pregnancy in Nigeria are rare; notably, second-hand smoking and smokeless tobacco have been hardly accounted for [23]. A survey from Enugu reported the prevalence of cigarette smoking as 4.5% [27]. Instead, most studies have focused on the general population, especially adolescents and young people [28, 29]. Some risk factors for tobacco use reported during pregnancy include younger age, lower educational levels, unplanned pregnancy, concurrent alcohol use, depression, stress and socialization [4, 15, 30]. Empirical evidence of these factors among the Nigerian pregnant population needs to be explored. Moreover, apart from assessing the alcohol and tobacco separately, it is also essential to evaluate the combined exposure to alcohol and tobacco because of the multiplicative effect on increasing perinatal risk [31].

There are few studies on alcohol consumption and tobacco use in pregnancy in Sub-Saharan Africa. However, there are projections of a significant rise in these behaviours in the coming decades [14, 15, 17]. This rise will negatively impact maternal and child wellbeing, which could reverse the gains from maternal and child mortality reduction efforts. Identifying the exposure to both alcohol and tobacco during pregnancy allows for interventions to reduce the risk of exposure in subsequent pregnancies. Unfortunately, very little attention has been given to the influence of alcohol consumption and tobacco exposure among pregnant women in Nigeria. Therefore, this study investigated the prevalence, pattern and risk factors of alcohol consumption and tobacco exposure among a cohort of pregnant women in Ibadan, Nigeria. The combined alcohol consumption and tobacco exposure during pregnancy and the associated factors were also examined. This study will provide further evidence required for providing policy-based interventions on alcohol and tobacco control, particularly among pregnant women in Nigeria.

## Methods

### Study setting and design

This study was conducted among a cohort of pregnant women in Ibadan, Nigeria – the Ibadan Pregnancy Cohort Study (IbPCS). The IbPCS is a prospective cohort study investigating pregnant women’s maternal obesity and lifestyle characteristics and their association with glycaemia, gestational weight gain and pregnancy outcomes within the Ibadan metropolis. The details of the methodology have been documented elsewhere [32]. In summary, pregnant women who reported for antenatal care at early gestation (≤ 20 weeks), who met the eligibility criteria and were willing to participate in the study, were recruited from four health facilities. The study setting was Ibadan, the capital city of Oyo State, southwest Nigeria from the four comprehensive obstetric facilities, which are the main referral centres for obstetric conditions within the Ibadan metropolis. These facilities were the University College Hospital, Adeoyo Maternity Teaching Hospital, Jericho Specialist Hospital and Saint Mary Catholic Hospital, Ibadan. The eligibility criteria included women ≤20 weeks’ gestation, aged ≥18 years, and women without severe medical complications. These women recruited early in pregnancy were followed up until delivery. Data were collected using pretested, interviewer-administered questionnaires and a desktop review of medical records. At enrolment, the research staff assessed maternal body mass index and lifestyle characteristics (including dietary pattern, physical activity, alcohol consumption, tobacco use during pregnancy, and sleep pattern). In this paper, alcohol consumption, tobacco use and combined exposure were the primary outcome variables, while sociodemographic, obstetrics and lifestyle factors were explanatory variables.

### Assessment of measures

The primary outcome variables, i.e. alcohol consumption and tobacco exposure, were obtained by self-reporting the presence or absence of the behaviour of interest. These variables were assessed in response to the following questions: 1.) Current smokers were those who answered yes to “Do you currently smoke cigarettes?” 2.) Overall tobacco exposure was obtained from current smokers, exposure to second-hand smoke (passive smokers, i.e. husbands currently smoke cigarettes) and those exposed to smokeless tobacco (Do you ingest any other forms of tobacco) 3.) Current alcohol users answered yes to “Do you currently take alcohol?” and was validated by the alcohol consumption on the food frequency questionnaire. The forms of alcohol consumed during this pregnancy were elicited in response to “Which type of alcoholic beverages have you taken in the last six weeks”. The factors associated with the combined exposure to alcohol and tobacco were also assessed. The household wealth index was constructed using household possessions or assets of study participants, e.g. radio, television, ownership of a house or means of transportation, source of drinking water, and type of toilet facility. The household data were transformed into scores and tertiles using factor analysis.

### Statistical analysis

Statistical analysis was performed using STATA version 13. Descriptive statistics were used to depict the study participants’ sociodemographic, obstetric and lifestyle characteristics by alcohol consumption and tobacco exposure. The prevalence of alcohol consumption, tobacco exposure, and the types of alcohol ingested were displayed graphically with bar graphs. The outcome variables in this study were alcohol consumption during pregnancy, tobacco exposure and the combined exposure to both alcohol and tobacco during pregnancy. The explanatory variables included maternal age, level of education, religion, marital status, monthly income, household wealth index, gravidity, parity, history of stillbirth, induced abortion, miscarriage, and contraceptive use. Bivariate logistic regression analyses were conducted to test the associations between the explanatory and the outcome variables. Variables significant at a 5% level of statistical significance at bivariate logistic analysis were subjected to multiple logistic regression analysis. The estimated unadjusted and adjusted odds ratios, 95% confidence intervals and *p*-values (*p* <  0.05) of associated factors were reported. Multicollinearity among the predictor variables in the multiple regression was tested by estimating the Variance Inflation Factor (VIF). A VIF ≥ 10 strongly indicates multicollinearity. For both multiple regression models, estimated VIF for all predictors ranged between 1.04–1.20 which confirmed the absence of multicollinearity among the predictor variables. Forest plots were used to help visualize the adjusted odds ratios from the multiple logistic regression analysis.

## Results

### Characteristics of pregnant women by alcohol consumption and tobacco exposure

The prevalence of alcohol consumption during pregnancy was 222 (12.7%). The prevalence of alcohol consumption during pregnancy was higher in younger women, single women and those with a lower level of education. For example, women with primary, secondary and tertiary education had a prevalence of 16.33, 12.9 and 12.46%, respectively. However, the prevalence of alcohol consumption was higher with increasing income, household wealth and parity. In addition, Christians reported a higher alcohol intake than Muslims (15.35% vs 9.09%). Women with histories of stillbirth (15.76%), induced abortion (24.91%) and tobacco exposure reported a higher alcohol intake during pregnancy (Table 1). The prevalence of tobacco exposure in the index pregnancy was 64 (3.7%). The prevalence of prenatal tobacco exposure was also higher in younger and unmarried women. The exposure to tobacco also increased with higher parity (nulliparous (3.42%); parity of 1–3 (3.85%); para ≥4 (4.21%). However, Muslims reported higher exposure to tobacco than Christians (4.13% vs 3.37%).

**Table 1:** Characteristics of pregnant women by alcohol consumption and tobacco exposure

Characteristics		Alcohol use status		Tobacco exposure	
	Total	Yes	No	Yes	No
**Overall prevalence**		**12.7%**		**3.7%**	
**Age**					
Less than 35 years	1389 (79.6)	180 (13.0)	1209(87.0)	52 (3.7)	1337 (96.3)
35 years and above	356 (20.4)	42 (11.8)	314 (88.2)	12 (3.4)	344 (96.6)
**Education**					
Primary or Less	49 (2.8)	8(16.3)	41(83.7)	2(4.1)	47 (95.9)
Secondary	504 (29.0)	65(12.9)	439(87.1)	22 (4.4)	482 (95.6)
Tertiary or Higher	1188 (68.2)	148(12.5)	1040(87.5)	39 (3.3)	1149(96.7)
**Religion**					
Christianity	1010 (58.2)	155(15.4)	855(84.6)	34 (3.4)	976 (96.6)
Islam	726 (41.8)	66(9.1)	660(90.9)	30 (4.1)	696 (95.9)
**Marital Status**					
Single	102 (6.3)	14(18.7)	88(86.3)	6 (5.9)	96 (94.1)
Married	1643 (93.7)	208(12.7)	1435(87.3)	58 (3.5)	1585 (96.5)
**Monthly Income (Naira)**					
Less Than 20,000	583 (38.0)	77 (13.2)	506 (86.8)	25 (4.3)	558 (95.7)
20,000-99,999	843 (55.0)	113 (13.4)	730 (86.6)	29 (3.4)	814 (96.6)
100,000 And Above	108 (7.0)	17 (15.7)	91 (84.3)	5 (4.6)	103 (95.4)
**Household Wealth**					
Poorest	583 (33.4)	60 (10.3)	523 (89.7)	21 (3.6)	562 (96.4)
Middle	580 (33.2)	75 (12.9)	505 (87.1)	20 (3.5)	560 (96.5)
Richest	582 (33.2)	87 (15.0)	495 (85.0)	23 (4.0)	559 (96.0)
***Obstetrics and lifestyle factors***					
**Parity**					
Nullipara	760 (43.7)	85(11.2)	675 (88 .8)	26(3.4)	734 (96.6)
1–3	882 (50.8)	122 (13.8)	760(86.2)	34 (3.8)	848 (96.2)
4 And Above	95 (5.5)	14 (14.7)	81(85.3)	4 (4.2)	91 (95.8)
**Gravidity**					
Primigravid	564 (32.4)	56(9.9)	508 (90.1)	15 (2.7)	549 (97.3)
2–4	983 (56.6)	128 (13.0)	855(87.0)	40 (4.0)	943 (96.0)
5 And Above	191 (11.0)	37 (19.4)	154 (80.6)	9 (4.7)	182 (95.3)
**BMI**					
Underweight	50 (3.0)	5 (10.0)	45 (90.0)	1 (2.0)	49 (98.0)
Normal Weight	845 (49.8)	102 (12.1)	743 (87.9)	30 (3.6)	815 (96.4)
Over Weight	473 (27.9)	56 (11.8)	417(88.2)	17 (3.6)	456 (96.4)
Obese	328 (19.3)	52 (15.8)	276 (84.2)	16 (4.9)	312 (95.1)
**History of stillbirth**					
Yes	165 (85.9)	26(15.8)	139 (84.24	9 (5.5)	156 (94.5)
No	1001 (14.1)	137 (13.7)	864(86.3)	40 (4.0)	961 (96.0)
**History of induced abortion**					
Yes	289 (25.0)	72 (24.9)	217 (75.1)	27 (3.1)	842 (96.9)
No	869 (75.0)	93 (10.7)	776 (89.3)	22 (7.6)	267 (92.4)
**History of miscarriage**					
Yes	386 (30.9)	51 (13.2)	335 (86.8)	35 (4.1)	827 (95.9)
No	862 (69.1)	124 (14.4)	738 (85.6)	15 (3.9)	371 (96.1)
**History of contraceptive use**					
Yes	287 (16.7)	43 (15.0)	244 (85.0)	15 (5.2)	272 (94.8)
No	1458 (83.3)	173 (12.1)	1258 (87.9)	47 (3.3)	1411 (96.7)
**Tobacco Exposure**					
Yes	64 (3.7)	17 (26.6)	47 (73.4)	–	–
No	1681 (96.3)	205 (12.2)	1476 (87.8)	–	–

### The prevalence of alcohol and tobacco use in pregnancy

**Fig. 1:**
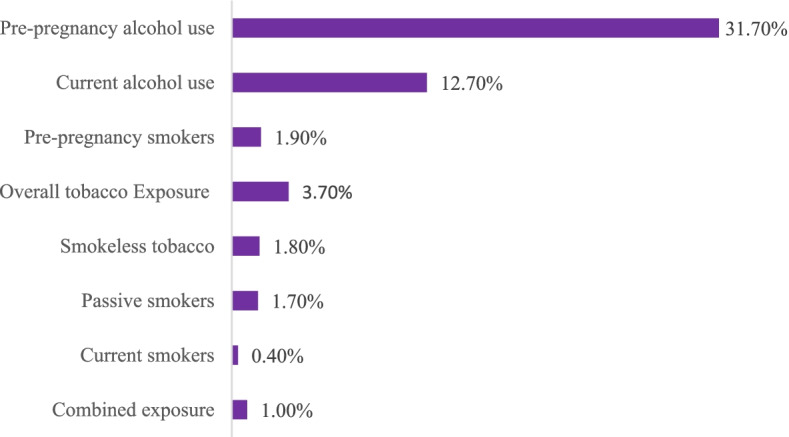
Prevalence of alcohol consumption, tobacco use and combined exposure among pregnant women in Ibadan, Nigeria

**Fig. 2:**
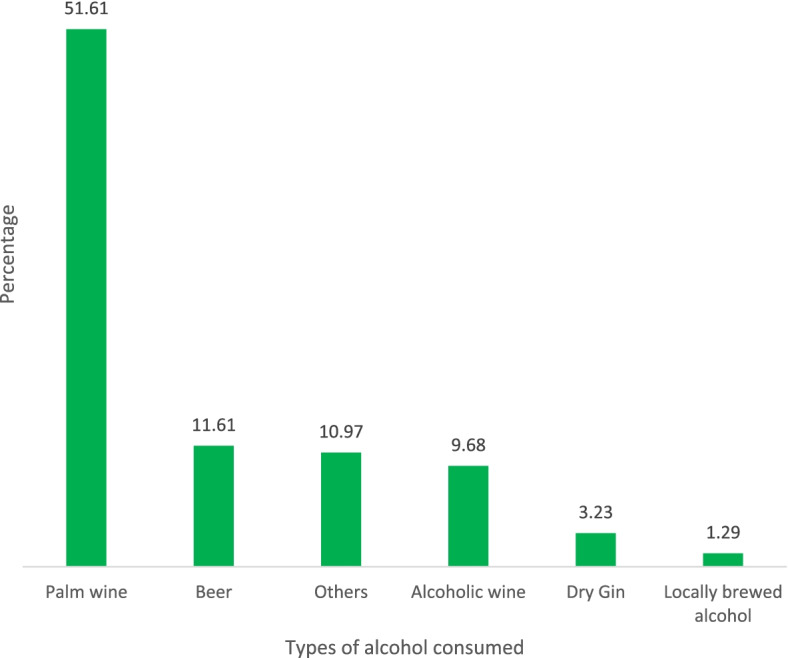
Types of alcohol ingested by pregnant women in Ibadan, Nigeria

The prevalence of alcohol and tobacco use in pregnancy is shown in Fig. 1. The prevalence of pre-pregnancy alcohol consumption and alcohol consumption during pregnancy were 551 (31.7%) and 222 (12.7%), respectively (one in every eight pregnancies is exposed to alcohol). Pre-pregnancy cigarette smoking was reported by 33(1.9%). The prevalence of tobacco exposure in the index pregnancy was 64 (3.7%), i.e. one in every 27 pregnancies is exposed to tobacco. In contrast, smoking, second-hand smoke and smokeless tobacco were 0.4%, 1.7% and 1.8%, respectively. The prevalence of combined exposure to alcohol and tobacco in pregnancy was 1.0%. Palm wine (52%) and beer (12%) were the most common forms of alcohol consumption among pregnant women (Fig. 2).

### Factors associated with alcohol consumption, tobacco use and combined exposure in pregnancy

**Fig. 3:**
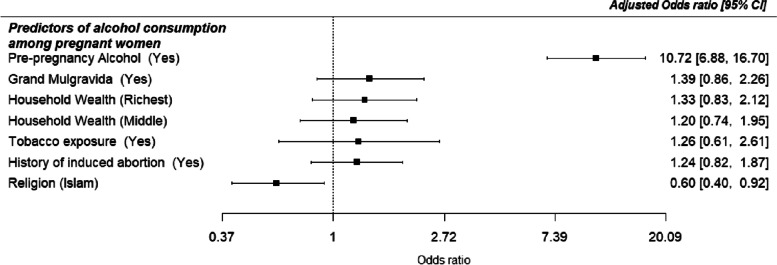
Forest plot showing the predictors of alcohol consumption among pregnant women in Ibadan, Nigeria

The factors associated with alcohol consumption are displayed in Table 2. The unadjusted logistic model showed that religion, household wealth, a history of induced abortion, tobacco exposure in the index pregnancy and pre-pregnancy alcohol use were significant factors associated with alcohol consumption in pregnancy. Specifically, Muslim women were less likely to consume alcohol during pregnancy [UOR = 0.55, 95% CI: 0.41–0.75, *p* <  0.001]. Women from rich households [UOR = 1.53, 95% CI: 1.08–2.18, *p* = 0.017) compared with women from poorer households. Also, women with a history of induced abortion had higher odds for alcohol intake [UOR =2.77, 95% CI: and 1.97–3.90, *p* < 0.001) than women without such a history. However, on multiple regression analysis, only religion, tobacco exposure during pregnancy and pre-pregnancy alcohol use remained significant. Muslims remained less likely to consume alcohol during pregnancy compared to Christians: [AOR = 0.60, 95% CI: and 0.40–0.92, *p* = 0.019). Pre-pregnancy alcohol use also increased the likelihood eleven times [AOR = 10.72, 95% CI: 6.88–16.70, *p* < 0.001) than women without prior alcohol experience. Forest plots displaying the adjusted odds ratios and 95% confidence intervals of the predictors of alcohol intake in pregnancy is presented in Fig. 3.

**Table 2:** Crude and Adjusted Odds Ratios and 95% Confidence Intervals of predictors of alcohol consumption among pregnant women in Ibadan, Nigeria

	Crude OR (95% CI)	*p*-value	Adjusted OR (95% CI)	*p*-value
**Characteristics**				
**Age**				
Less than 35	1			
35 and above	0.90 (0.63–1.29)	0.558		
**Education**				
Primary or less	1			
Secondary	0.76 (0.34–1.69)	0.500		
Tertiary or higher	0.73 (0.34, 1.59)	0.426		
**Religion**				
Christianity	1		1	
Islam	0.55 (0.41–0.75)	**< 0.001**	0.60 (0.40–0.92)	**0.019**
**Marital status**				
Single	1			
Ever married	0.91 (0.51–1.63)	0.754		
**Income (monthly income in naira)**				
Less than 20,000	1			
20,000-99,999	1.02 (0.75–1.39)	0.914		
100,000 and above	1.23 (0.69–2.17)	0.481		
**Household wealth**				
Poorest	1		1	
Middle	1.29 (0.90–1.86)	0.161	1.20 (0.74–1.95)	0.455
Richest	1.53 (1.08–2.18)	0.017	1.33 (0.83–2.12)	0.238
**Parity**				
Nullipara	1			
1–3	1.28 (0.95–1.71)	0.108		
4 and above	1.37 (0.74–2.53)	0.309		
**BMI**				
Not obese	1			
Obese	1.39 (0.99–1.95)	0.055		
**History of stillbirth**				
No	1			
Yes	1.18 (0.75–1.86)	0.478		
**History of induced abortion**				
No	1		1	
Yes	2.77 (1.97–3.90)	**< 0.001**	1.24 (0.82–1.87)	0.141
**History of miscarriage**				
No	1			
Yes	0.91 (0.64–1.29)	0.581		
**Tobacco exposure**				
No	1		1	
Yes	2.60 (1.47–4.62)	**0.001**	1.26 (0.61–2.61)	0.613
**Pre-pregnancy alcohol use**				
No	1		1	
Yes	12.02 (8.49–17.02)	**< 0.001**	10.72 (6.88–16.70)	**< 0.001**
**Grand multigravida**				
No	1		1	
Yes	1.78 (1.20–2.63)	**0.004**	1.39 (0.86–2.26)	0.179

**Fig. 4:**
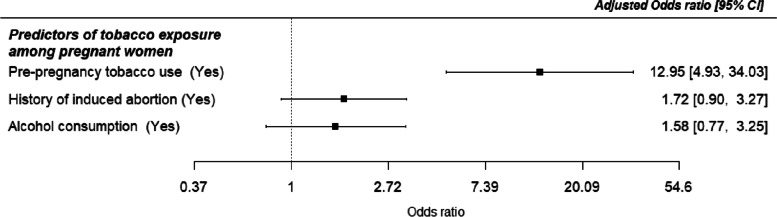
Forest plot showing the predictors of tobacco exposure among pregnant women in Ibadan, Nigeria

The factors associated with tobacco among pregnant women are shown in Table 3. The unadjusted logistic model showed that a history of induced abortion, alcohol consumption in the index pregnancy and pre-pregnancy tobacco use were significant factors associated with tobacco exposure in pregnancy. Specifically, women with a history of induced abortion had higher odds of tobacco exposure [UOR =2.57, 95% CI: 1.44–4.59, *p* = 0.001) than women who had never procured an abortion. Alcohol consumption also increased the odds of tobacco exposure during pregnancy [UOR =2.60, 95% CI: 1.47–4.62, *p* = 0.001). However, with multiple logistic regression analysis, only pre-pregnancy tobacco use was the only factor associated with tobacco exposure after adjusting for other confounders, with about 13 times higher odds [AOR = 12.95; 95% CI: 4.93, 34.03, *p* < 0.001). Forest plots displaying the adjusted odds ratios and 95% confidence interval of the predictors of tobacco exposure in pregnancy is presented in Fig. 4.

**Table 3:** Crude and Adjusted Odds Ratios and 95% Confidence Intervals of predictors of tobacco exposure among pregnant women in Ibadan, Nigeria

Characteristics	Crude OR (95% CI)	*p*-value	Adjusted OR (95% CI)	*p*-value
**Age**				
Less than 35	1			
35 and above	0.898 (0.63–1.29)	0.543		
**Education**				
Primary or less	1			
Secondary	1.073 (0.25–4.70)	0.926		
Tertiary or higher	0.798 (0.19–3.40)	0.760		
**Religion**				
Christianity	1			
Islam	1.237 (0.75–2.04)	0.404		
**Marital status**				
Single	1			
Ever married	0.59 (0.25–1.39)	0.225		
**Monthly income in naira)**				
Less than 20,000	1			
20,000-99,999	0.80 (0.46–1.37)	0.410		
100,000 and above	1.08 (0.41–2.90)	0.873		
**Household wealth**				
Poorest	1			
Middle	0.96 (0.51–1.78)	0.887		
Richest	1.10 (0.60–2.01)	0.754		
**Parity**				
Nullipara	1			
1–3	1.13 (0.67–1.90)	0.641		
4 and above	1.24 (0.42–3.64)	0.694		
**BMI**				
Not obese	1			
Obese	1.41 (0.79–2.52)	0.245		
**History of stillbirth**				
No	1			
Yes	1.39 (0.66–2.91)	0.389		
**History of induced abortion**				
No	1		1	
Yes	2.57 (1.44–4.59)	**0.001**	1.72 (0.90–3.27)	0.099
**History of miscarriage**				
No	1			
Yes	0.96 (0.52–1.77)	0.885		
**Alcohol consumption**				
No	1		1	
Yes	2.60 (1.47–4.62)	**0.001**	1.58 (0.77–3.25)	0.215
**Pre-pregnancy tobacco use**				
No	1		1	
Yes	11.26 (5.00–25.37)	**< 0.001**	12.95 (4.93–34.03)	**< 0.001**
**Grand-multigravida**				
No	1			
Yes	1.34 (0.65–2.76)	0.425

Table 4 displays the prevalence of combined exposure to alcohol and tobacco use (CEATU) and the associated factors among pregnant women in Ibadan, Nigeria. The prevalence of combined alcohol and tobacco use exposure was 1.0% (i.e. one in every hundred women). The only factor significantly associated with CEATU was a history of induced abortion [UOR = 4.91; 95% CI: 1.6–15.2, *p* = 0.006).

**Table 4:** Prevalence of combined exposure to alcohol and tobacco, odds ratios and 95% confidence intervals of the associated factors among pregnant women in Ibadan, Nigeria

Characteristics	Combined exposure to alcohol and tobacco			
	Yes	No	UOR (95% CI)	*p*-value
**Overall Prevalence**	**17 (1.0%)**	1728 (99.0%)		
**Age**				
Less than 35	17 (1.2)	1372 (98.8)	1	
35 and above	0 (0.0)	356 (100.0)		
**Education**				
Primary or less	0 (0.0)	49 (100.0)	1	
Secondary	5 (1.0)	499 (99.0)	1.07 (0.37–4.70)	0.926
Tertiary	11 (0.9)	1177 (99.1)	1	
**Religion**				
Christianity	12 (1.2)	998 (98.8)	1	
Islam	5 (0.7)	721 (99.3)	1.237 (0.75–2.04)	0.404
**Marital status**				
Single	2 (2.0)	100 (98.0)	1	
Married	15 (0.9)	1628 (99.1)	0.46 (0.10–2.04)	0.308
**Monthly income in naira)**				
Less than 20,000	6 (1.0)	577 (99.0)	1	
20,000-99,999	9 (1.1)	834 (98.9)	1.04 (0.37–2.93)	0.944
100,000 and above	1 (0.9)	107 (99.1)	0.9 (0.11–7.54)	0.922
**Household wealth**				
Poorest	7 (1.2)	576 (98.8)	1	
Middle	4 (0.7)	576 (99.3)	0.57 (0.17–1.96)	0.374
Richest	6 (1.0)	576 (99.0)	0.86 (0.29–2.57)	0.783
**Parity**				
Nullipara	10 (1.3)	750 (98.7)	1	
1–3	7 (0.8)	875 (99.2)	1.13 (0.67–1.90)	0.641
4 and above	0 (0.0)	95 (100.0)	1.24 (0.42–3.64)	0.694
**BMI**				
Not obese	13 (1.0)	1355 (99.0)	1	
Obese	4 (1.2)	324 (98.2)	1.29 (0.42–3.97)	0.661
**History of stillbirth**				
No	11 (1.1)	990 (98.9)	1	
Yes	2 (1.2)	163 (98.8)	1.10 (0.24–5.03)	0.898
**History of induced abortion**				
No	5 (0.6)	864 (99.4)	1	
Yes	8 (2.8)	281 (97.2)	4.91 (1.60–15.2)	**0.006**
**History of miscarriage**				
No	13 (1.5)	1355 (99.0)	1	
Yes	1 (0.3)	385 (99.7)	1.70 (0.02–2.30)	0.088

## Discussion

Reducing tobacco and harmful alcohol is one cornerstone of achieving the 2030 Agenda for Sustainable Development Goals (SDGs). Both substances directly impact the health-related SDGs, which seek to ensure healthy lives and promote wellbeing for all ages and indirectly on the other goals [2]. Prenatal alcohol consumption and tobacco exposure which are largely preventable, have adverse effects on maternal and child wellbeing. These issues have received scant attention in developing countries, including Nigeria. This study investigated the prevalence, pattern and predictors of alcohol consumption and tobacco exposure among pregnant women in Ibadan, Nigeria. The prevalence of alcohol use during pregnancy among the study participants was 12.7% which is high considering that there is no safe limit for alcohol consumption during pregnancy. In a recent systematic review, the pooled prevalence of alcohol intake during pregnancy in sub-Saharan Africa was 15.7% [14]. Under-reporting alcohol use may not be ruled out due to social desirability bias and religious constraints. Notably, our finding was much lower than estimates within and outside Nigeria, 36.3% in Geneva, Switzerland [33] [34], 37.1% in Addis Ababa, Ethiopia [35] and 37.6% in Accra, Ghana [36], South-South Nigeria 59.29% [16]. The variation across this region could be due to contextual and socio-cultural differences and measurement issues. Researchers have observed that the rise in alcohol consumption in Africa is due to economic growth, increasing social acceptability of the habit, and changing gender roles [14]. This projection underscores the need for public interventions, including creating awareness and providing health education on the harmful effect of alcohol consumption among pregnant women.

Sociodemographic factors have been strongly associated with alcohol consumption in pregnancy in both developing and developed countries. For example, studies within Nigeria and Africa [15–17] found that women who consumed alcohol were generally younger, less educated and not married. Conversely, studies from developed countries reported that alcohol consumption during pregnancy was commoner among older women and well educated [37, 38]. This study did not demonstrate any statistically significant difference in the sociodemographic characteristics between women who consumed and did not drink alcohol during pregnancy. This lack of significance by sociodemographic factors may be due to a pervasive culture that frowns against alcohol use among African women, especially during pregnancy [14].

Pre-pregnancy alcohol consumption was a significant predictor of alcohol consumption in this current study. Pregnant women with a history of alcohol use were eleven times more likely to consume alcohol during pregnancy. These findings are corroborated by other studies from South-East Nigeria [17], Tanzania [39], Ethiopia [35] and Uganda [40]. The most likely reason could be that those women who consumed alcohol during pregnancy have developed tolerance to alcohol and have difficulty discontinuing the use because of withdrawal symptoms [41]. More importantly, most pregnancies are unplanned. The actual prevalence of prenatal alcohol exposure is closer to the pre-pregnancy values than the values detected at the assessment visit, which occurs much later in pregnancy and most likely reflects the number of women with continuing use. Also, alcohol consumption during pregnancy was significantly associated with religion, as pregnant Muslims were less likely to consume alcohol (AOR = 0.60) than Christians. This observation was similar to other findings [16, 42]. This association could be because of the strict Islamic condemnation of alcohol consumption. Additionally, women who had tobacco exposure during pregnancy also had an increased likelihood of alcohol ingestion.

Palm wine was our study population’s most common alcoholic beverage (about 50%). Palm wine is an alcoholic beverage produced from the sap of palm trees, it's high in sugars and has an alcoholic content of about 5% [43]. It is common in West African and South Asia countries during social celebrations and festivities [44, 45]. Its consumption among pregnant women may be due to the belief that it promotes lactation and has beneficial medicinal effects. However, pregnant women should be discouraged from ingesting palm wine because of its alcoholic content which is harmful to the fetus. The high sugar content may also be detrimental to pregnant women who are obese or at risk of hyperglycaemia. The high chemical contaminants are also a possible food safety hazard for pregnant women [46]. There is no safe level of alcohol use during pregnancy, therefore the WHO recommends that health care providers should ask all pregnant women about their use of alcohol as early as possible in the pregnancy and at every antenatal visit [6].

The prevalence of tobacco exposure during pregnancy in this study was 3.7%, but cigarette smoking, second-hand smoke and smokeless tobacco were 0.4, 1.7 and 1.8%, respectively. The finding supports the low level of cigarette smoking reported in Africa [24, 25] Pre-pregnancy tobacco use was the only significant predictor of tobacco exposure in our study that was associated with tobacco exposure during pregnancy. Previous studies showed that most women who smoked cigarettes or tobacco before pregnancy ended up smoking during pregnancy [47]. Notably, 1% of our study population, i.e. one in every hundred pregnancies, had a combined exposure to alcohol and tobacco. Women exposed to tobacco during pregnancy had a higher odds of alcohol consumption among our study participants. This double exposure to tobacco and alcohol increases the risk of stillbirth [48], sudden infant death syndrome (an important cause of post-neonatal mortality) [49] and other adverse outcomes more than each substance alone [50]. Additionally, we found that a history of induced abortion increased the likelihood of CEATU by fivefold compared with women without a similar history. Studies have reported that a history of induced abortion and other high-risk behaviours increases the risk of depression among pregnant women and, consequently, prenatal substance use [51–53].

Williams et al. [54] demonstrated a low-cost community intervention strategy for reducing prenatal alcohol exposure in the Congo. Such interventions may also successfully control prenatal alcohol and tobacco in the Nigerian setting. Although, there has been a considerable silence on alcohol, tobacco and the combined use among pregnant women by the public and stakeholders in Nigeria. This is due to a lack of awareness of the magnitude of the problem. Unfortunately, there is a projected increase in prenatal alcohol use and tobacco exposure in LMIC, including Nigeria [1, 14]. Therefore, this study provides the needed information for stakeholders (obstetricians, gynaecologists, midwives) and policymakers on controlling alcohol and tobacco among pregnant women in Nigeria. The policy interventions should include creating awareness, educating women on the associated health hazards and adverse pregnancy outcomes during antenatal care, and formulating national guidelines by adopting international frameworks on alcohol and tobacco. The WHO guidelines for preventing and managing tobacco use and second-hand smoke exposure in pregnancy recommend effectively screening pregnant women for their past and present use of tobacco during antenatal care. They should provide interventions for tobacco cessation and advice on the risk of exposure to second-hand smoke [55].

This study’s main strength is its contribution to a much neglected maternal health hazard - alcohol consumption and tobacco exposure – and the associated factors among the Nigerian pregnant population using a reasonably large sample. Additionally, the combined effects and the associated factors were also examined. The use of multiple health facilities also enhanced the generalizability of the study. This study also provides the required evidence for the policy interventions in Nigeria. However, the study has its limitation. First, the assessment of the outcome variables was based on self-report, which is prone to measurement bias from under-porting, social desirability and recall bias. Also, the study assessed the occurrence of alcohol consumed, but there was a lack of a quantitative measure of alcohol. Hence binge drinking could not be assessed. Therefore the use of validated tools is recommended for further research. Additionally, two important predictors (pre-pregnancy alcohol use and pre-pregnancy cigarette smoking) had wide confidence intervals indicating uncertainty from a relatively small sample size in relation to a rare outcome like tobacco exposure hence future studies should use a much larger sample size.

## Conclusions

Alcohol consumption and tobacco exposure were not uncommon among our study population; indicating that they are an emerging threat to maternal and child health in Nigeria. This finding may be conservative given the possibility of social desirability. Control policy and programmes should be implemented to prevent the use among pregnant and reproductive-age women in Nigeria, especially during antenatal care. Factors to be targeted in programme implementation include pre-pregnancy alcohol consumption and tobacco exposure, religion and history of high-risk behaviours, including a history of induced abortion.

## Data Availability

The datasets generated and analysed during the current study are not publicly available because they contain potentially identifying and confidential information but are available from the corresponding author on reasonable request if they meet the criteria for accessing confidential data.
